# In vitro toxicity assessment of bioavailable iron in coal varieties of Central India

**DOI:** 10.1371/journal.pone.0309237

**Published:** 2024-09-19

**Authors:** Ruchika Kishor Jain, Anshumali, Prasad Sherekar, Amit Nayak, Shraddha Jaiswal, Komal Pimpalghare, Rajani Tumane, Aruna Jawade, Shubhangi Kailas Pingle, Sanvidhan G. Suke, Rajpal Singh Kashyap, Bibhuti Bhusan Mandal

**Affiliations:** 1 Department of Environmental Science and Engineering, Indian Institute of Technology (Indian School of Mines), Dhanbad, Jharkhand, India; 2 Department of Biochemistry, National Institute of Miners’ Health, Nagpur, Maharashtra, India; 3 Department of Biotechnology, Priyadarshini College of Engineering, Nagpur, Maharashtra, India; 4 Research Laboratory, G.M. Taori, Central India Institute of Medical Sciences (CIIMS), Nagpur, Maharashtra, India; 5 Department of Biochemistry, Regional Occupational Health Centre (Southern), Bengaluru, Karnataka, India; 6 Department of Mining Engineering, Indian Institute of Technology (IIT) Kharagpur, Kharagpur, West Bengal, India; University of Kalyani, INDIA

## Abstract

**Introduction:**

Information on bioavailable Iron (BAI) content in respirable coal dust (RCD) is crucial to address occupational health and safety, especially in preventing coal workers’ pneumoconiosis (CWP).

**Materials and methods:**

In the present study, we determined BAI concentrations in seventy-seven coal samples collected from ten coal mining regions of Central India. The cytotoxic potential of BAI-RCD was established invitro by using alveolar epithelial (A549) and macrophage (U937) cell lines. The oxidative/antioxidant status, inflammations, and genotoxicity attributed to BAI-RCD exposure were evaluated and correlated with CWP pathophysiology.

**Results:**

The mean BAI concentrations in the coal samples (n = 77) range from (275 to 9065 mg kg^-1^) and showed wide variability. Both cell lines were exposed to low (275 mg kg^-1^), moderate (4650 mg kg^-1^), and high (9065 mg kg^-1^) BAI-RCD samples showed significant (*p* < 0.001) cytotoxicity in a dose-dependent manner (low < moderate < high) compared to the control. After BAI-RCD treatment, both cell lines showed a decrease in antioxidant stress measures (SOD, CAT, and GSH) and a significant (*p* < 0.001) increase in oxidative stress parameters (NADPH, MPO, LPO, and PC). Furthermore, these cell line models demonstrated a statistically significant (*p* < 0.001) dose-dependent increase in cytokines (TGF-β1, IL-1β, TNF-α, MCP-1, and IL-6 cytokines) and oxidative DNA damage marker (8-OH-dG).

**Conclusion:**

Results indicated that the central India coals (even at low BAI content) may be accountable for inflammatory responses and cytotoxicity. Hence, BAI can be important characteristic to establish safety standards for coal dust exposure before active mining.

## 1. Introduction

Coal is the most viable source of energy worldwide [1], with the stigma of emissions of particulate matter such as dust and aerosols from coal mining. After China, Coal India Limited (CIL) is the world’s largest producer of coal and one of the biggest employers of corporate workers [2]. The National Institute for Occupational Safety and Health (NIOSH) recommended exposure levels of coal dust 1 mg/m^3^ and 3 mg/m^3^ for 10 hours/day for the USA and Indian mines, respectively [3]. Long-term occupational exposure to hazardous coal dust causes significant lung damage, respiratory disorders, and even death [4]. Among lung diseases, coal workers’ Pneumoconiosis (CWP) has received considerable attention due to its high fatality rates [5, 6] and resulted in 25,000 deaths globally in 2013 [7]. China has recorded 915,000 cases of occupational pneumoconiosis cumulatively by the end of 2021. Coal miners are one of the primary demographic groups afflicted by occupational pneumoconiosis, with around 450,000 individuals surviving the condition. This suggests that coal miners’ pneumoconiosis is now one of the most deadly and prevalent occupational diseases in China [8]. In India, the prevalence of CWP in coal workers was 3.03%, ranging from 1.52% to 4.76% in different coal mining areas [9]. Although coal is not a fibrogenic agent, other minerals found in coal dust can affect lung architecture and contribute to the development of CWP [10, 11]. The active agent within coal appears to be Iron in coal dust particles, but not quartz, after noticing the characteristics of coal dust responsible for CWP [12, 13].

Iron is the most well-known transition metal, involved in various physiological activities (oxygen transport, oxidative metabolism, cell proliferation, etc.) [14]. Bioavailable Iron (BAI) is defined as the Iron (both Fe^2+^ and Fe^3+^) released in 10 mM phosphate solution at pH 4.5, which mimics the phagolysosomes of cells [15–17]. However, not all iron species in the coal are bioavailable for oxidant formation and cause adverse health effects [18]. BAI is mainly sourced from pyrite, sulfuric acid, and total iron chemical interactions and primarily contains water-soluble Iron, such as ferrous and ferric sulfate, which can be initially present in coal or obtained by oxidizing pyrite (FeS_2_), acid solubilization of siderite (FeCO_3_) or ferrous silicate (FeSiO_3_) [12].

**Fig 1:**
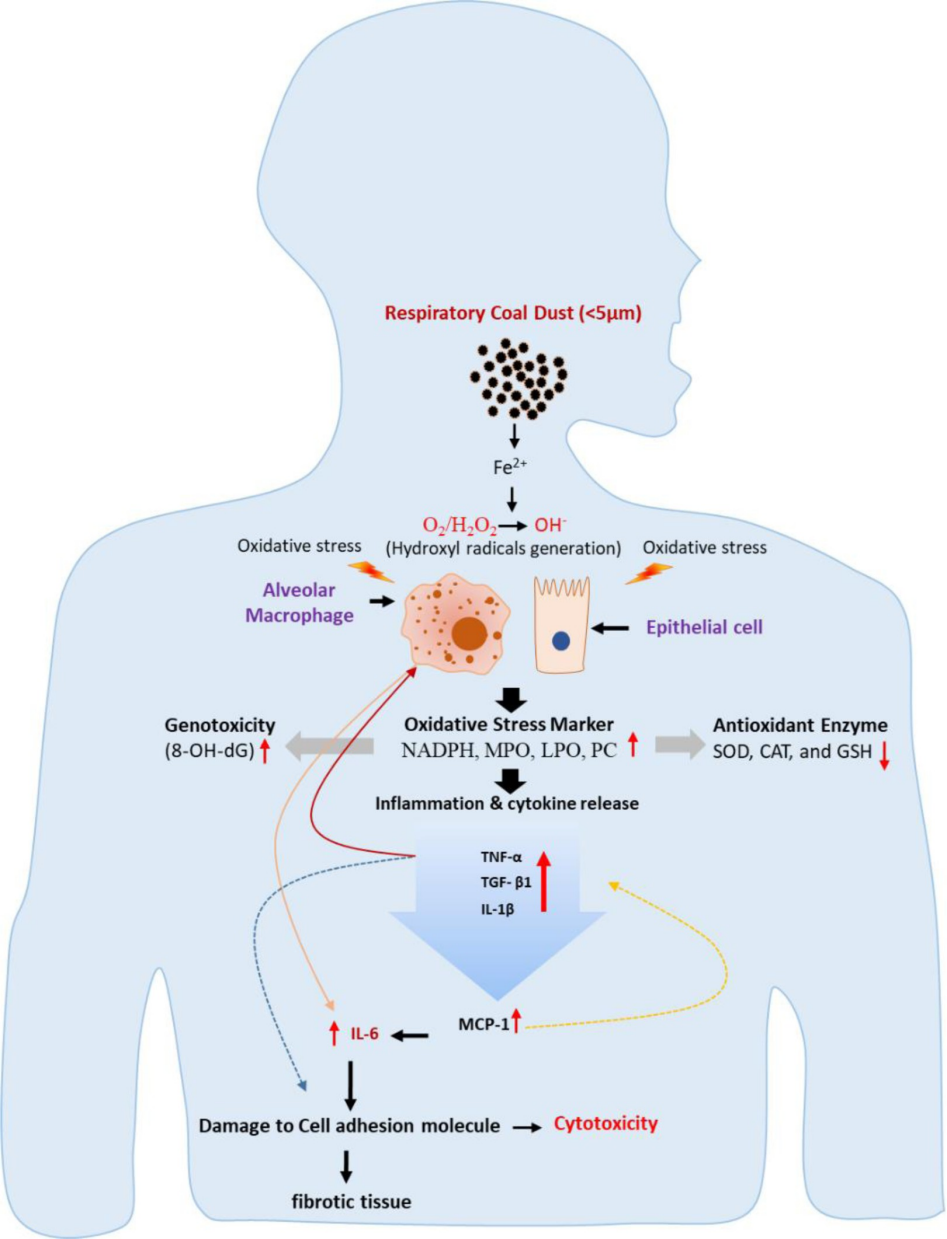
Mechanism of BAI in respirable coal dust to induce cytotoxicity.

Inhaled BAI-rich coal dust is absorbed by resident alveolar or interstitial macrophages and epithelial cells, triggering an inflammatory cascade [19] and resulting in alveolitis [20] followed by reparative and fibrotic stages, which lead to overexpression of fibronectin and collagen, causing fibrosis [16] (Fig 1). Human lung cells lines such as A549, U937, and THP-1 have previously been used *in vitro* to recreate immunopathological events of BAI-containing coal dust exposure in the lungs, such as BAI stress-induced cytokines and fibrosis formation [16, 21–23]. Macrophages derived from U937 monocyte cells help in modeling immunological interactions, which are important targets for the emergence of disease caused by respirable particles. [24, 25]. Oxidative stress and inflammatory indicators played a crucial part in the pathogenesis of Pneumoconiosis [26].

Interestingly, the concentrations of BAI in different types of Indian coal and in-vitro experimental data are currently unknown. The current research aimed to determine the BAI and elemental compositions of coal samples collected from ten coal mine regions of central India. An appropriate in vitro model was developed using epithelial A549 and U937 macrophage cell lines to assess its cytotoxic effects on CWP. This work will offer evidence that levels of BAI in the coals may be applied as a predictor of coal’s toxicity applicable before mining. Possibly this pointed out to better strategic planning for screening and prevention programs that carefully monitor early adverse effects and, thus reduce health care costs related to the coal use. There are limited studies available on the pneumoconiosis caused by the presence of BAI in the coal dust particles, till date there is no Indian data in this regard. Therefore, it is an urgent need for such study in India.

## 2 Materials and methods

### 2.1 Chemical reagents

RPMI 1640 Medium (Roswell Park Memorial Institute 1640 Medium) containing 1X Antibiotic antimycotic solution (Gibco Life Technology), Fetal Bovine Serum (FBS) (Gibco Life Technology), Trypsin-EDTA Solution (0.025% Trypsin and 0.01% EDTA) (Himedia India), phorbol-12-myristate-13-acetate (PMA) (Sigma, USA)

### 2.2 Collection of coal samples

**Fig 2:**
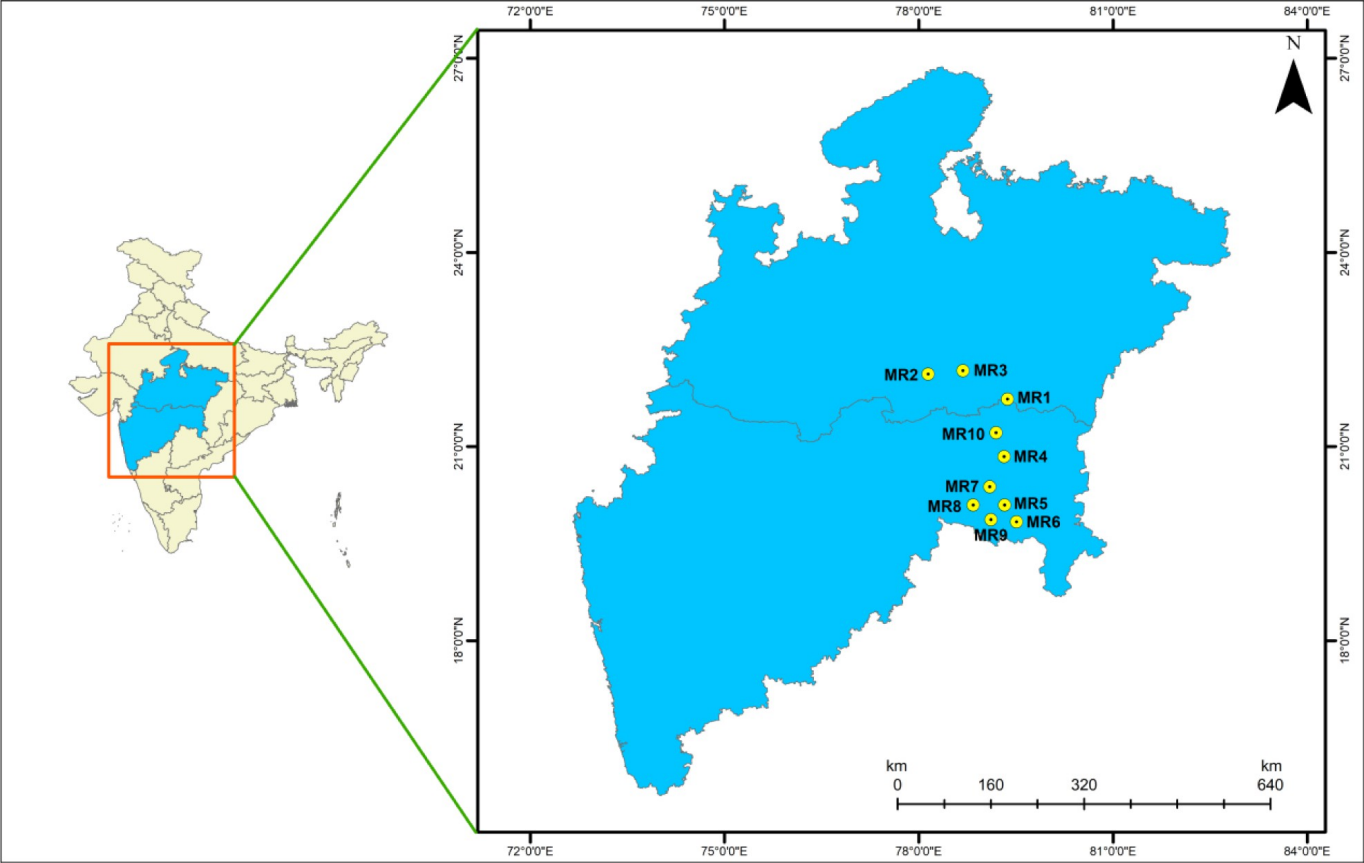
Map of Central India showing locations of coal sample collection from ten mining regions (MR). Each dot represents a mine location of coal sample collection. The map was created using the ArcGIS 10.4 version from a GIS student. Map used from open source https://onlinemaps.surveyofindia.gov.in/Home.aspx.

Seventy-seven coal samples (n = 77) were collected from thirty open cast mines (OC) and thirty-two underground mines (UG) located in ten coal mining regions of central India (Fig 2). A written agreement was obtained from each coal mine authority to collect coal samples. Five kg of hard coal samples were taken from the seam faces (n = 40), conveyor belt (n = 5), bunker stock (n = 4), surface belt (n = 4), and surface bunker (n = 16) areas of mines and transported to the laboratory using air-tight polyethylene bags (HiDispo Bag-14, Himedia). The representative samples were prepared using coning and quartering (criss-cross) methods [27]. The sample codes were given to individual reference samples according to their collection region.

### 2.3 Preparation of respirable coal dust (RCD)

Coal samples were pulverized using a ball mill (S. K. Mitra Coal Inspection Private Ltd. at Nagpur) and then passed through a vibrating cup mill (Herzog, Hungary) to obtain respirable particle size (< 10 μm) followed by conventional sieving. A laser particle size analyzer (Saturn Digitizer II 5205) was used to confirm that more than 75% of coal particles had a diameter size of less than 10 μm S1 Table and S1–S3 Figs. Prepared RCD samples were stored in desiccators to avoid moisture for further use.

### 2.4 Analysis of bioavailable iron in respirable coal dust (BAI-RCD)

An iron assay kit (QuantiChromTM, USA) was used to measure BAI content (Fe^3+^and Fe^2+^) with certain modifications [28]. Briefly, 10 mg of RCD was suspended in 5 mL of 1M sodium bicarbonate solution (pH 8.2) and incubated at 80°C for 30 minutes in a water bath (Remi RSB-12). The clear supernatant was utilized to determine BAI after centrifuging at 3000 rpm for 5 min (Remi C-24 plus). A supernatant ion reducer converted Fe^3+^ to Fe^2+^, and chromogen created blue colour with both free and reduced Fe^2+^. The BAI concentrations in coal samples were directly proportional to the color intensity observed at 590 nm. The slope was obtained by plotting the kit’s standard graph (S4 Fig) of known iron concentrations. The BAI concentration was determined by Eq (1).


$$BAI\;(\mathrm{mg}\;{\mathrm{kg}}^{‐1})=\frac{\mathrm{OD}\;\mathrm{sample}-\mathrm{OD}\;\mathrm{Blank}}{\mathrm{Slope}}$$ (1)

Further, the BAI present in coal samples (S2 Table) were classified into low (> 1000 mg kg^-1^), moderate (1000–7000 mg kg^-1^), and high (> 7000 mg kg^-1^) BAI-containing coal categories [5, 12].

### 2.5 Analysis of BAI-RCD at acidic pH

For BAI release in acidic conditions using the standard protocol [16], one representative sample from each category, i.e., low (B3), moderate (J8), and high (D6), with some modifications, was carried out. 1 g of coal dust was suspended in 15 mL of falcon tube with 10 mL of 10 mM phosphate solution at pH 4.5. The tube was placed at room temperature on a rotary shaker (100 rpm/min), and 1 mL of aqueous coal suspension was consecutively taken at 3 hours, one day, three days, and seven days after incubation. Coal particles were separated by centrifuging suspensions at 6000 rpm for 10 min. The BAI released (Fe^2+^ and Fe^3+^) from coal in supernatants was measured by the previously explained iron assay kit procedure.

### 2.6 Stability of BAI

The stability of BAI released from the coal was evaluated for different periods at basic pH, with two sets of coal sample suspension (10 mg RCD in 5 ml of 1 M sodium bicarbonate pH 8.2) [16]. The first suspension set was incubated at room temperature on a rotary shaker. The suspension was removed and heated at 80°C in a water bath for 30 minutes after 3 hours, one day, three days, and seven days (incubation before heating; condition A). The second set was heated in a water bath at the same time and temperature as the first suspension, then incubated for 3 hours, one day, three days, and seven days (incubation after heating; condition B). An iron assay kit determined the BAI released in both treatment conditions and analyzed its stability.

### 2.7 Elemental composition of coal

For the analysis of the total concentrations of Iron (Fe) and other trace elements Nickel (Ni), Cobalt (Co), Chromium (Cr), Arsenic (As), Copper (Cu), Calcium (Ca), and silicon dioxide (SiO_2_), by ICP-OES (iCAP 7400 ICP-OES Duo model, Thermo Scientific, USA), the representative coal samples (B3, J8, and D6) were prepared using the classical wet chemical digestion method [29]. The coal samples were dried in a hot air oven at 105°C for 1 hour to remove moisture content. After drying, samples were cooled in a desiccator, weighed 0.5 g, and transferred into a 250 mL beaker. The samples were moistened with double distilled water, and 25 mL of concentrated HCl and 5 mL of concentrated HNO_3_ were added. The mixture of coal and acids was heated on the hot plate for 30–45 minutes, then cooled at room temperature, diluted up to 50 mL with double distilled water, and filtered using Whatman no. 42 filter paper in a 100 mL standard calibrated flask to obtain a clear solution for the analysis. The ultra-pure chemicals were used to avoid foreign impurities during the digestion and extraction of the coal samples.

### 2.8 Cell culture

Human alveolar basal epithelial cell line A549 (adherent cell line) and human alveolar monocytic cell line U937 were procured from National Centre for Cell Science (NCCS), Pune, India. Cells were maintained in RPMI 1640 media containing 10% fetal bovine serum (FBS) and 1X antibiotic anti-mycotic solution and were passage every 3 to 4 days.

#### 2.8.1 Differentiation of U937 cell line

The U937 cells were cultivated at 1×10^6^ cells/ml density and treated with 10, 50, and 100 ng/ml doses of phorbol-12-myristate-13-acetate (PMA). To eliminate excess PMA and non-adherent cells, cells were washed twice with ice-cold PBS after 24 hours. The adhering cells were kept alive for another 48 hours (recovery phase). The morphology and attachment of differentiated U937 (U937^d^) cells were examined using 0.5% crystal violet staining under an inverted phase-contrast microscope (Invitrogen Evos XL Core, USA). The differentiated cells were used to create an in vitro macrophage cell line model.

#### 2.8.2 In vitro model of CWP

**Fig 3:**
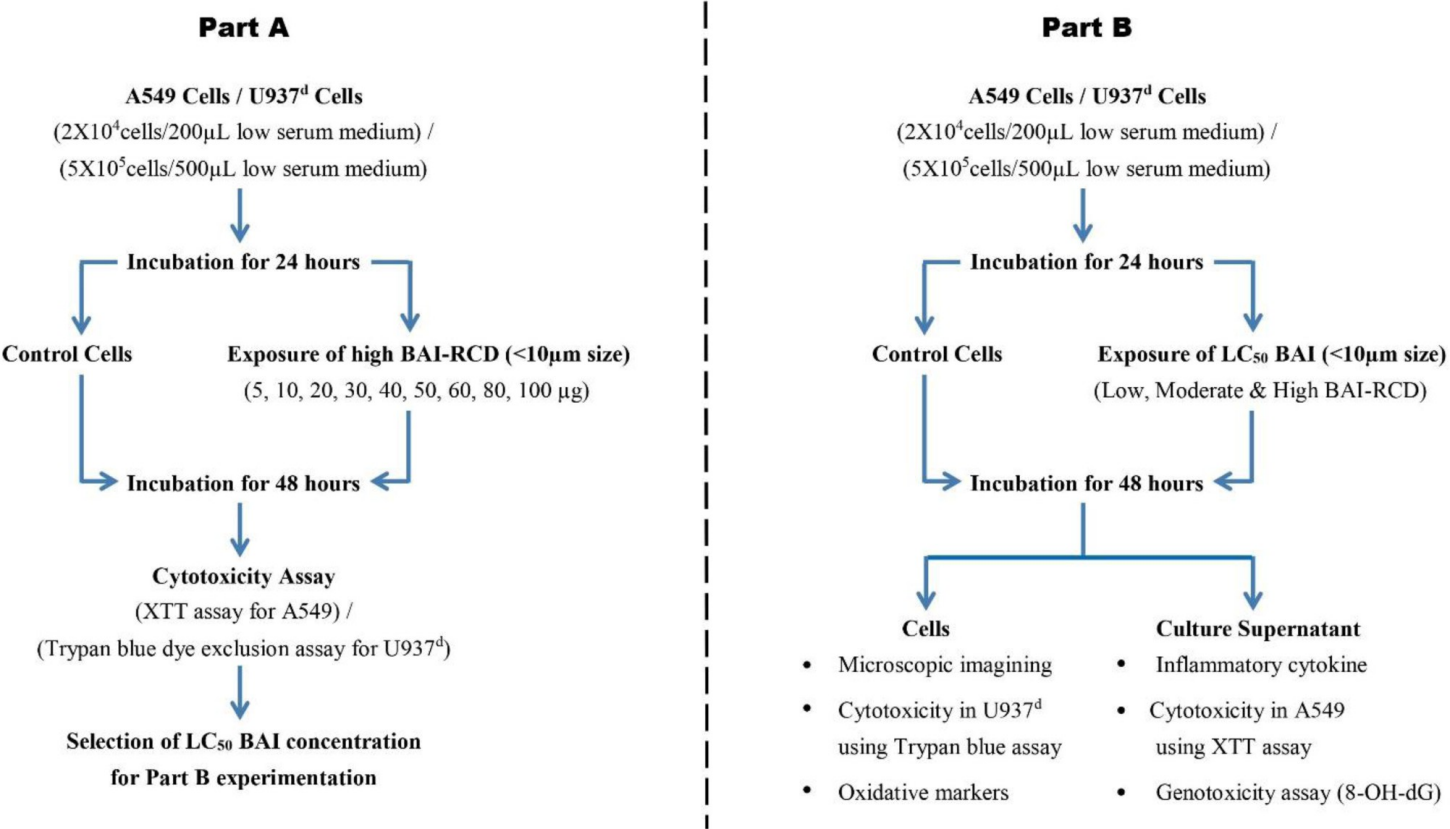
Experimental workflow for calculating LC_50_ of BAI-RCD in A549 and U937^d^ cell lines (Part A) and comparative evaluation of CWP induction potential of low, moderate, and high BAI-containing RCD in both cell lines (Part B).

Each experiment included plates with untreated cells as a negative control. The high-range BAI RCD sample (D6) was chosen for *in vitro* exposure toxicity analysis in A549 and U937^d^ cell lines [5, 12]. 48-well cell culture plates were seeded with 2×10^4^ A549 cells/200 μL. U937^d^ cells were seeded at 5×10^5^ cells/ mL on 24 cell culture plates. Both cells were cultivated for 24 hours. After culturing, the medium was changed to a low serum medium (2% FBS) in the absence (control) and presence of different doses of RCD having the BAI concentrations (45 ng/5 μg, 90 ng/10 μg, 180 ng/20 μg, 270 ng/30 μg, 360 ng/40 μg, 450 ng/50 μg, 540 ng/60 μg, 720 ng/80 μg, and 900 ng/100 μg, w/w of RCD). These cells were cultured for another 48 hours (Fig 3 part A). XTT and trypan blue dye exclusion assays measured A549 and U937^d^ cell cytotoxicity [30]. Regression analysis calculated BAI’s 50% cytotoxic lethal concentration (LC_50_). In both cell lines, the fatal doses of high-BAI-containing RCD (LC_50_), which causes 50% cytotoxicity, were utilized to compare the CWP induction potential of low and moderate and high-BAI RCD samples (Fig 3 part B). U937^d^ Cells were stained with 0.25% crystal violet stain after the experimental endpoint for 10 minutes. After staining, cells were washed with PBS 3 to 5 times, air dried, and examined under a phase contrast inverted microscope (Invitrogen Evos XL Core, USA).

#### 2.8.3 Cytotoxicity assessment

At the experimental endpoint, 20 μL of working XTT solution [2,3-bis-[2-methoxy-4-nitro-5-sulfophenyl]-2H-tetrazolim-6-carboxanilide inner salt, 1mg/ml and PMS (N-methyl dibenzopyrazine methyl sulfate, 0.5mg/ml)] was added to wells. The plate was incubated for another four hours. After incubation, a microplate reader (Robonik Readwell Touch, India) assessed optical density (OD) at 450 nm. The BAI-induced toxicity from the reaction’s color reduction, assuming 100% control cell viability, was determined by Eqs (2) and (3).


$$\%\;\mathrm{Viability}=\frac{\mathrm{Corrected}\;\mathrm{OD}\;\mathrm{of}\;\mathrm{sample}}{\mathrm{Control}\;\mathrm{OD}}\times 100$$ (2)

Toxicity=100‐%Viability (3)

At the experimental endpoint, the U937^d^ cells were washed twice with RPMI and trypsinized (without EDTA) to remove excess coal particles. At low spin, each well’s cells were centrifuged in a complete RPMI medium in an eppendorf tube. Cells were treated for 5 min at RT with 0.4% trypan blue in PBS to evaluate cytotoxicity. A cell counter (BioRad TC10 automated cell counter, India) counted live and dead cells.

### 2.9 Oxidative stress measurement

Oxidative stress markers such as nicotinamide adenine dinucleotide phosphate oxidase (NADPH), myeloperoxidase (MPO), lipid peroxidase (MDA), protein carbonyl (PC), and antioxidants, including superoxide dismutase (SOD), catalase (CAT), and reduced glutathione (GSH) were measured using enzyme linked immunosorbent assay (ELISA) kit purchased from Genexbio Health Sciences. The readings were taken at 450 nm within 10 minutes, and calculations were made using the standard graph (Standardization data provided in S5 Fig).

### 2.10 Measurements of cytokines and genotoxicity parameters

After experimentation, cell culture supernatants were collected and stored at– 80°C. The release of transforming growth factor-β1 (TGF-β1), interleukin-1β (IL-1β), tumor necrosis factor-α (TNF-α), monocyte chemotactic protein-1 (MCP-1), and interleukin-6 (IL-6) into the cell culture supernatants were measured using the ELISA based kit purchased from Bioassay Technology Laboratory, Shanghai, China, as per manufacturers instruction. The concentration of evaluated cytokines was represented in pg/ml (Standardization data provided in S6 Fig).

Genotoxicity was measured by estimating the level of 8-hydroxydeoxyguanosine (8-OH-dG) in the cell culture supernatant after 48 hours, using the ELISA kit method as directed by the manufacturer (Bioassay Technology Laboratory, Shanghai, China). The absorbance was measured at 450 nm using a microplate reader. The concentration of 8-OH-dG was reported in ng/L. (Standardization data provided in S7 Fig).

### 2.11 Statistical analysis

All the numerical data were represented as mean ± SD. One-way analysis of variance (ANOVA) was used to compare means among control, low, medium, and high BAI concentrations. Tukey’s HSD test was used for *post-hoc* analysis, and statistical significance was examined at *p* < 0.05. Data were considered highly significant if *p* < 0.01 or *p* < 0.001. All analyses were performed using SPSS version 20 (IBM CORP, ARMONK, USA).

## 3 Results

### 3.1 BAI classification

The seventy-seven coal samples from ten coal mining regions showed wide variability in mean BAI concentrations, ranging from 275 mg kg^-1^ to 9065 mg kg^-1^. The MR 4 and MR 2 showed the highest and lowest BAI mean values, respectively (Table 1).

**Table 1:** The mean concentration of BAI in ten coal mine regions (MR) of Central India.

Mine Regions	Sample Codes	No. of Samples	BAI Range	Mean BAI (mg kg^-1^) ± SD
MR 1	A1 to A16	16	2277–4292	3452 ± 550.7
MR 2	B1 to B6	6	275–3800	1383 ± 1259.3
MR 3	C1 to C6	6	375–2675	1583 ± 936.3
MR 4	D1 to D8	8	1270–9065	4077 ± 2680.6
MR 5	E1 To E10	10	475–4875	1369 ± 1471.3
MR 6	F1 to F8	8	1800–3850	2920 ± 752.5
MR 7	G1 to G4	4	3225–4466	3805 ± 620.5
MR 8	H1 to H4	4	1525–2585	2021 ± 493.9
MR 9	I1 to I5	5	2375–4905	3051 ± 1059.7
MR 10	J1 to J10	10	800–4650	2505 ± 1007.1
		**n = 77**	**275–9065**	**2679 ± 1513.2**

The BAI concentrations were grouped into three different BAI ranges: low (< 1000 mg kg^-1^), moderate (1000–7000 mg kg^-1^), and high (> 7000 mg kg^-1^). The two coal samples (3%) showed high concentrations of BAI, while sixty-three coal samples (82%) were recorded in the moderate range and twelve coal samples (15%) in the low range (Table 2).

**Table 2:** Details of coal samples classification based on BAI concentrations.

Range of BAI	BAI concentration (mg kg^-1^)	No. of samples *(*%) (Total n = 77)	Sample code
Low	< 1000	12 (15)	B2, B3, C4, C6, E1, E3 to E8, J2
Moderate	1000–7000	63 (82)	A1 to A16, B1, B4 to B6, C1 to C3, C5, D1 to D4, D7, D8, E2, E9, E10, F1 to F8, G1 to G4, H1 to H4, I1 to I5, J1, J3 to J10
High	> 7000	2 (3)	D5, D6

The B3 (275 mg kg^-1^), J8 (4650 mg kg^-1^), and D6 (9065 mg kg^-1^) were selected as low, moderate, and high BAI category coal samples to understand the alterations in A549 and U937 cell lines deciphering invitro CWP (Table 3).

**Table 3:** Selected representative coal samples based on BAI concentration for exposure studies.

Range of BAI	Mine region	Sample code	BAI concentration (mg kg^-1^)
Low	MR 2	B3	275
Moderate	MR 10	J8	4650
High	MR 4	D6	9065

### 3.2 BAI release under acidic pH

The B3, J8, and D6 were tested for BAI release at pH 4.5 and expressed as a percent release of Fe (Table 4). There was a significant increase in the release of BAI from 0.47 to 22.18%, 0.92 to 26.17%, and 2.74 to 54% in the low, moderate, and high BAI-RCD coal samples under the three hours to three days of incubation, respectively. However, all representative BAI-RCD coal samples showed significant decreases in the BAI released after seven days of incubation.

**Table 4:** BAI as a percent release at pH 4.5.

BAI -RCD samples	BAI release in mg kg^-1^ (%) from RCD at different incubation time			
	3 Hours	Day 1	Day 3	Day 7
Low (B3)	1.31 (0.47)	26 (9.45)	61 (22.18)	43 (15.63)
Moderate (J8)	4.3 (0.92)	170 (3.65)	1217 (26.17)	70 (1.5)
High (D6)	249 (2.74)	2395 (26.42)	4895 (54)	4287 (47.29)

*Note—Released BAI were parts per million (mg kg^-1^) of coal (w/w); (% release)–released BAI percent against total BAI concentration in mg kg^-1^ of coal

### 3.3 BAI stability at pH 8.2 under different conditions (A & B)

The BAI stability test results of the B3, J8, and D6 under conditions A and B are given in Table 5. In Condition A, all BAI-RCD samples were incubated for various durations before being heated for BAI analysis. After 3 hours, the release of BAI by B3 samples was 95.27%; it started declining after seven days of incubation up to 71.63%. All samples had a similar trend (for J8 95.05–75.33; D6 96.61–83.07). The D6 sample appeared relatively stable compared to J8 and B3; its BAI release percentage decreased less throughout various incubations. In condition B, all BAI-RCD samples were heated first and then incubated for different durations to assess BAI release. It was found that when all of the samples were heated simultaneously, the BAI released at the end of the incubation period was dramatically reduced.

**Table 5:** BAI stability at pH 8.2 under different treatment conditions (A & B).

BAI -RCD samples	BAI stability in mg kg^-1^ (%) at different incubation time			
	*Condition A*: *Incubation before heating*			
	3 Hours	Day 1	Day 3	Day 7
Low (B3)	262 (95.27)	240 (87.27)	229 (83.27)	197 (71.63)
Moderate (J8)	4422 (95.05)	4050 (87.09)	3941 (84.75)	3503 (75.33)
High (D6)	8758 (96.61)	8429 (92.98)	8210 (90.56)	7531 (83.07)
	*Condition B*: *Incubation after heating*			
Low (B3)	153 (55.63)	131 (47.63)	65 (23.63)	2 (0.72)
Moderate (J8)	1773 (38.12)	1160 (24.94)	372 (8.00)	43 (0.92)
High (D6)	3765 (41.53)	2364(26.078)	875 (9.65)	175 (1.93)

*Note—Released BAI were parts per million (mg kg^-1^) of coal (w/w); (% release)–released BAI percent against total BAI concentration in mg kg^-1^ of coal.

### 3.4 Metal compositions of coal

Metal concentrations correlated with BAI RCD samples are depicted in Table 6. The D6 (high) sample showed high concentrations of Ni (37.98 mg kg^-1^), Co (22.14 mg kg^-1^), As (11.19 mg kg^-1^), Fe (10.23%), and Ca (0.45%) as compared to J8 (moderate) and B3 (low) samples. However, the concentrations of Cr, Cu, and SiO_2_ were higher in both J8 and B3 BAI-RCD samples than in the D6 sample.

**Table 6:** Metals concentrations in selected coal samples.

Coal samples	Concentration in mg kg^-1^					Concentration in %		
	Ni	Co	Cr	As	Cu	Fe	Ca	SiO_2_
B3	12.22	7.99	19.48	<1.0	8.53	0.44	0.11	9.1
J8	28.59	10.35	22.02	0.63	13.3	0.5	0.3	8.3
D6	37.98	22.14	11.9	7.19	8.18	10.23	0.45	7.66

*Note–Levels of Ni, Co, Cr, As, and Cu were reported in parts per million (mg kg^-1^) of coal (w/w); levels of Fe, Ca, and SiO_2_ were reported in percentage (%).

### 3.5 Effects of BAI-RCD on A549 cells

**Fig 4:**
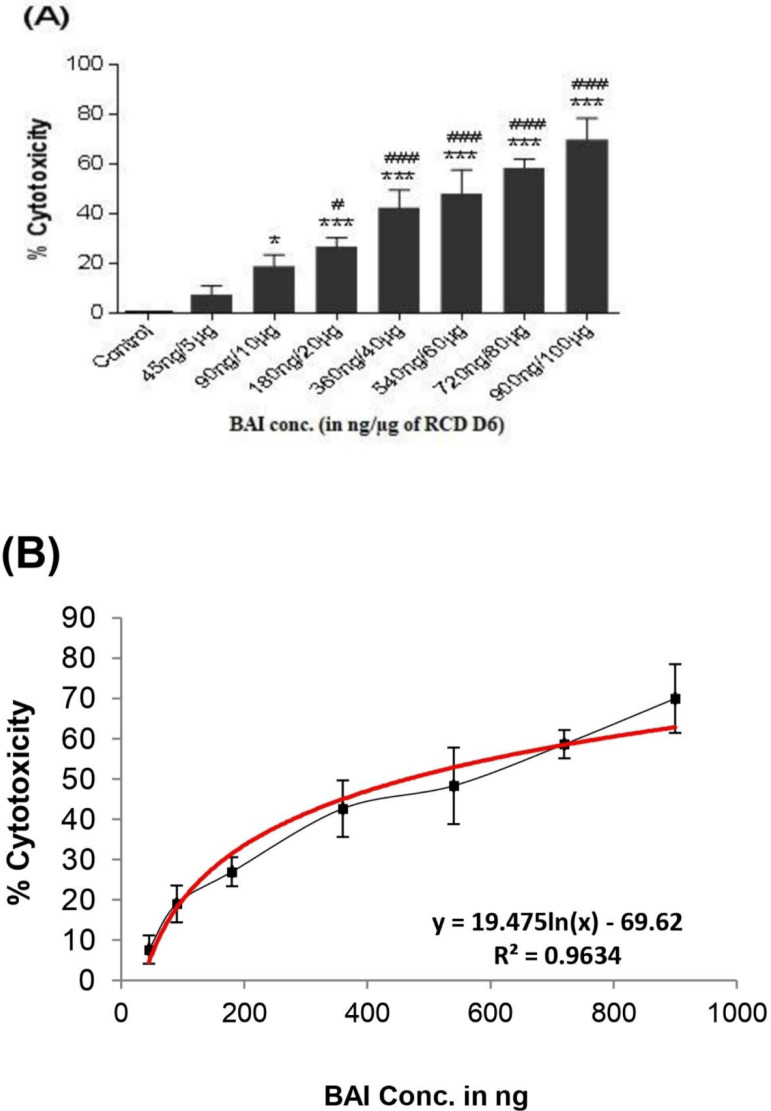
(A) Cytotoxic effects of increasing BAI concentration of RCD (D6) on A549 cell line using XTT assay. (B) Standard plot of cytotoxicity used for LC_50_ calculation of BAI-RCD in A459 cells exposed to RCD-D6 sample of high BAI. The data are presented as mean ± SD of 4 individual experiments in triplicate. Cytotoxic response presented as the percentage of cell viability relative to controls. (*p < 0.05, ***p < 0.001vs. control, ^#^*p* < 0.05, ^###^*p* < 0.001 vs lowest BAI concentration 45ng/5μg of RCD).

The dose-dependent cytotoxicity was observed in the A549 cells exposed to increasing doses of the BAI-RCD-D6 sample (Fig 4A). The BAI-RCD dose (BAI concentration in ng/ μg of RCD D6 sample) of 90 ng / 10 μg (*P < 0.05) and 180 ng / 20 μg (***p < 0.001) showed significantly higher cytotoxicity than the control. Moreover, 27%, 43%, 48%, 59%, and 69% of cytotoxicity attributed to doses greater than 180 ng /20 μg, significantly different from the dose of 45 ng / 5 μg of RCD. The LC_50_ of BAI induced 50% cytotoxicity in A549 cells was found to be 465 ng / 51 μg of RCD D6 sample (Fig 4B).

### 3.6 Effects of BAI-RCD on U937 cells

**Fig 5:**
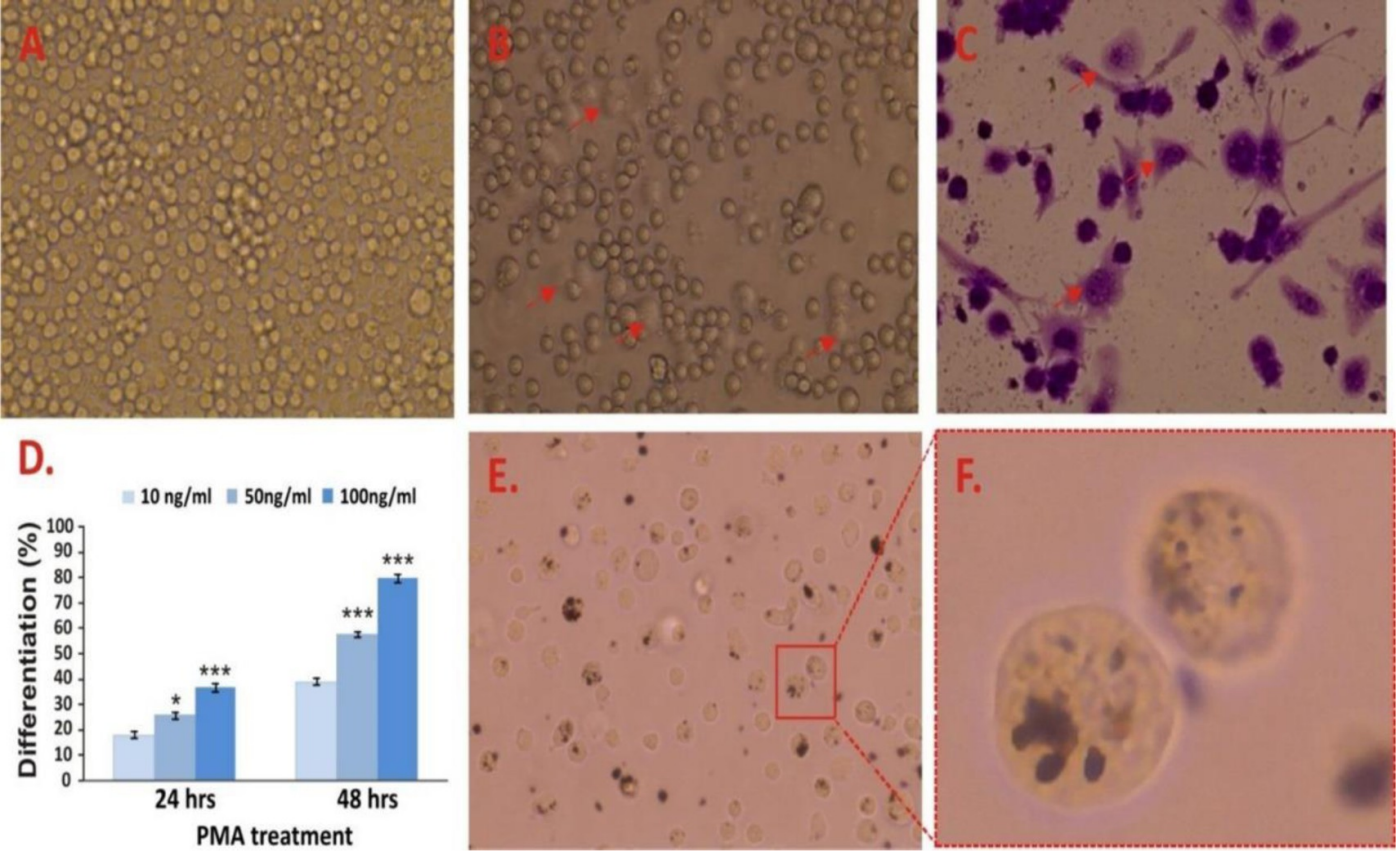
(A) Microscopic image of undifferentiated monocytic U937 cells. (B) Differentiated macrophage U937^d^ cells with PMA (100ng/ml). (C) Differentiated cells stained with crystal violet. (D) Dose and time dependent differentiation of U937 cell after induction with different PMA concentrations for 24 and 48 hours. (E & F) Engulfed RCD particles by U937^d^ macrophages. Data expressed as mean ± SD of three replicates. Significantly correlated with PMA (10ng/mL): *p < 0.05, ***p < 0.001; Tukey’s HSD test.

**Fig 6:**
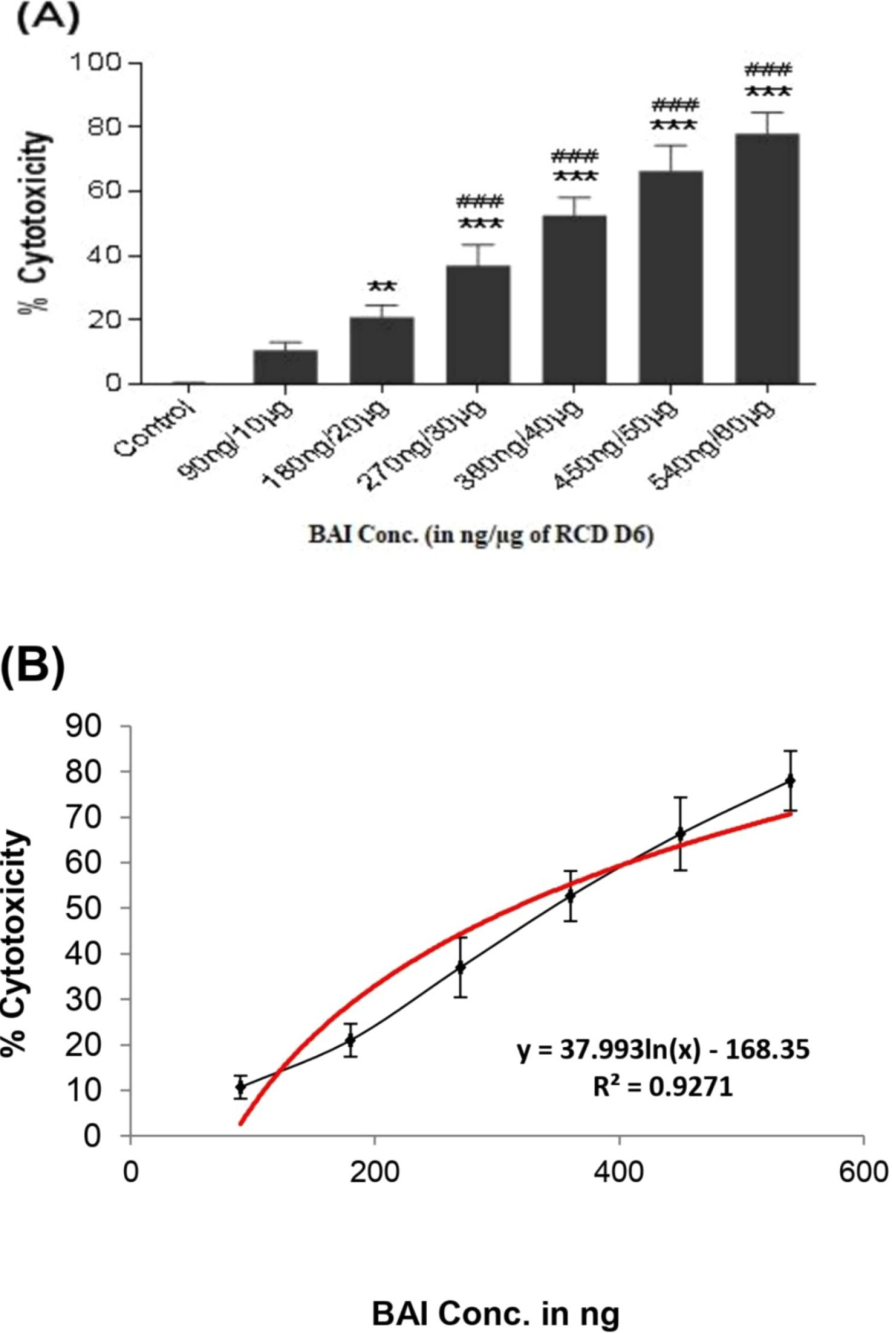
(A) Cytotoxic effects of increasing BAI concentration of RCD (D6) on U937^d^ cell line using trypan blue dye exclusion assay. (B) Standard plot of cytotoxicity used for LC_50_ calculation of BAI-RCD in U937^d^ cells exposed to RCD-D6 sample of high BAI. The data are presented as mean ± SD of 4 individual experiments in triplicate. Cytotoxic responses are presented as the percentage of cell viability relative to controls: (*p< 0.05, ***p< 0.001vs. control, ^###^*p*< 0.001vs. lowest BAI concentration 90ng/10μg of RCD).

Differentiation in macrophages to become adherent cells was further confirmed by flat and extensive, with dense cells, huge vacuolized or foamy, spindle-shaped cells, and cells with pseudopodia (Fig 5A–5C). The 80% percentage of differentiation of U937 cells (U937^d^) was observed with stimulation by 100 ng / mL of PMA for 48 hours (Fig 5D). The exposed RCD particles were engulfed by differentiated U937 macrophages (Fig 5E and 5F). Similar to A549, U937^d^ exhibits dose-dependent cell cytotoxicity after exposure to all doses of BAI-RCD (*p <0.05, ***p < 0.001) compared to the control. Moreover, amplified (^###^ *p* < 0.001) cytotoxicity was noted for the dose of BAI concentration of 270 ng / 30 μg of RCD and above against 180 ng / 20 μg BAI-RCD dose (Fig 6A). However, 35 μg of RCD containing 313 ng of BAI was shown to cause 50% cytotoxicity (LD_50_) in U937^d^ cells (Fig 6B).

### 3.7 Evaluation of cytotoxicity in both A549 and U937^d^ cell lines

**Fig 7:**
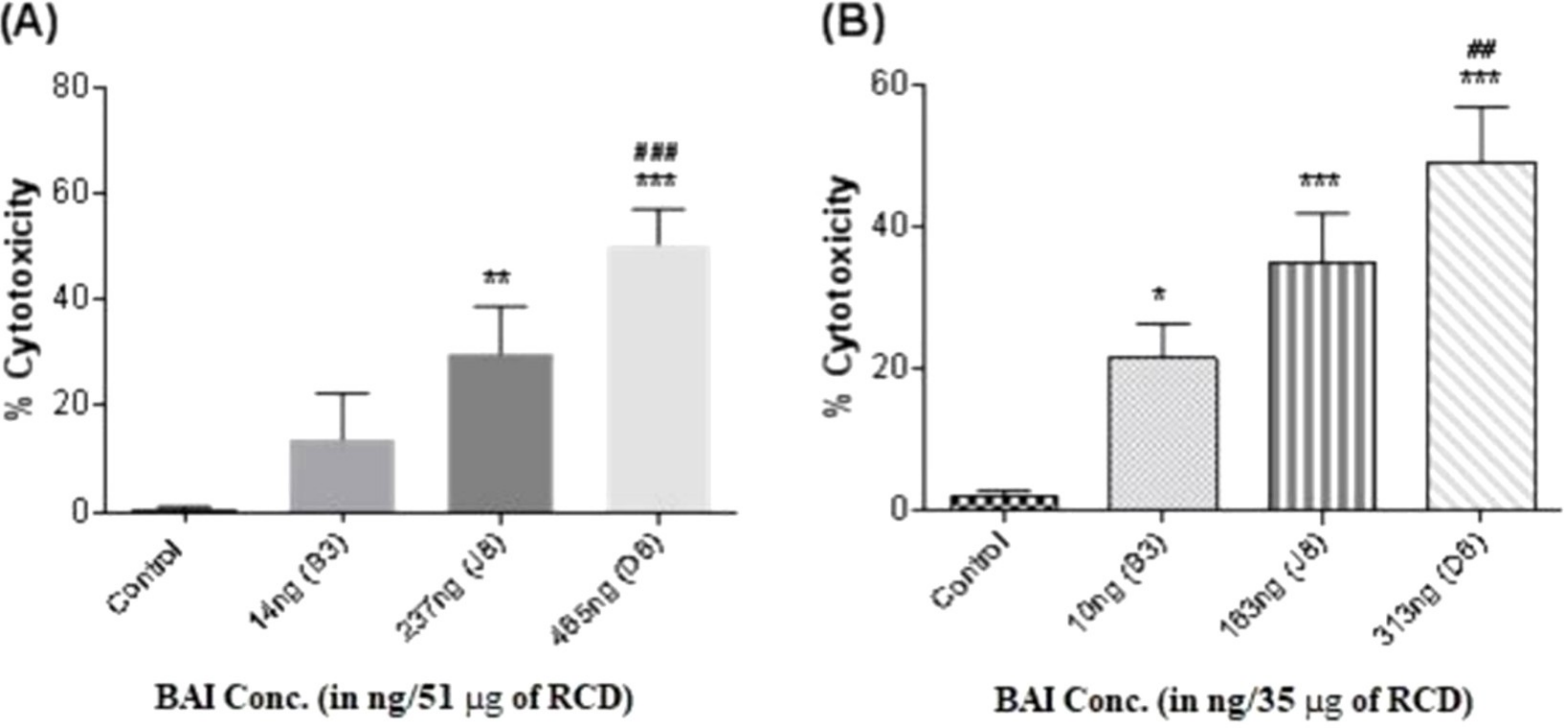
The effects (% cytotoxicity) of various BAI-containing RCD samples on (A) A549 and (B) U937^d^ using XTT and trypan blue assays. The data are presented as mean ± SD of 4 individual experiments in triplicate. Cytotoxic responses are presented as the percentage of cell viability relative to controls: (*p< 0.05, **p< 0.01, and***p< 0.001vs. control, ^###^*p*< 0.001vs.low BAI-RCD-B3 sample).

The A549 and U937^d^ cells were further exposed with respective fixed volumetric dose (LC_50_) of RCD-containing different BAI concentrations (i.e., for A549: B3 = 14 ng / 51 μg, J8 = 237 ng / 51 μg, and D6 = 465ng / 51 μg and for U937 B3 = 10 ng / 35 μg, J8 = 163 ng / 35 μg, and D6 = 313 ng / 51 μg). The J8 and D6 RCD samples showed (Fig 7A) more significant toxicity (*p* < 0.01 and *p* < 0.001) in A549 cells compared to the control. Whereas only D6 RCD has a significant difference (*p* < 0.001) against the B3 RCD sample. Similarly, the higher toxicity (*p* < 0.001) in U937^d^ cells was attributed due to J8 and D6 RCD samples (Fig 7B). Correspondingly, the D6 RCD sample also showed elevated cytotoxic effects (*p* < 0.001) compared to B3 RCD containing low BAI concentrations.

### 3.8 Evaluation of oxidative and antioxidant status in both A549 and U937^d^ cell lines

The results of oxidative stress markers and antioxidants level in A549 and U937^d^ cells exposed to representative RCD samples were shown in Table 7. As compared to the control, all studied parameters demonstrated significant differences (*p* < 0.05, *p* < 0.01, and *p* < 0.001) for exposed samples in A549 cells except LPO and CAT levels attributed due to low BAI-RCD. The status of oxidative and antioxidant parameters in U937^d^ was significantly different (*p* < 0.05 and *p* < 0.001) for exposed BAI-RCD samples related to control, irrespective of the PC content attained for the low and moderate BAI-RCD. In both cell lines, High BAI-RCD revealed a significant increase in NADPH, MPO, LPO, and PC and a substantial decrease in antioxidants containing SOD, CAT, and GSH levels.

**Table 7:** Comparison of mean oxidative stress and antioxidant status across BAI-RCD exposed A549 and U937^d^ cells.

Oxidative stress markers	Samples	A549	U937^d^
NADPH (ng/L)	Control	160.3 ±3.5	210.7 ± 4.7
	Low	174.2 ± 4.5*	358.8 ± 5.2***
	Moderate	235.0 ± 6.2***	395.8 ± 4.2***
	High	241.3 ± 4.5***	423.5 ± 5.0***
MPO (ng/L)	Control	242.7 ± 5.0	264.0 ± 2.6
	Low	304.3 ± 3.8***	275.0 ± 2.0*
	Moderate	316.3 ± 5.0***	303.3 ± 2.5***
	High	343.7 ± 3.1***	311.7 ± 7.0***
LPO (nmol/mL)	Control	3.17 ± 0.59	5.09 ± 1.20
	Low	5.07 ± 0.45	18.24 ± 1.53***
	Moderate	6.59 ± 0.98**	18.28 ± 0.86***
	High	18.57 ± 1.06***	32.34 ± 2.48***
PC (ng/mL)	Control	13.5 ± 0.7	8.8 ± 1.0
	Low	22.2 ± 0.9***	9.9 ± 0.3
	Moderate	25.6 ± 0.8***	9.7 ± 1.0
	High	34.3 ± 1.2***	18.1 ± 1.2***
SOD (ng/L)	Control	1430 ± 20	1797 ± 50
	Low	873 ± 15***	1267 ± 15***
	Moderate	933 ± 23***	910 ± 46***
	High	820 ± 26***	753 ± 30***
CAT (KU/L)	Control	42.2 ± 1.7	52.1 ± 2.7
	Low	39.8 ± 0.6	46.5 ± 1.7*
	Moderate	30.4 ± 1.8***	44.9 ± 0.7**
	High	26.7 ± 2.7***	40.0 ± 2.0***
GSH (ng/mL)	Control	67.4 ± 2.5	96.9 ± 2.0
	Low	58.4 ± 1.9**	64.5 ± 1.5***
	Moderate	56.4 ± 2.0**	58.2 ± 1.3***
	High	53.9 ± 2.9***	52.2 ± 2.0***

*Note-The data are presented as mean ± SD of three replicates. Significant correlation with control: (*P< 0.05

**P< 0.01

***P< 0.001; Tukey’s test).

### 3.9 Evaluation of cytokines and DNA damage marker (8-OH-dG) in both A549 and U937^d^ cell lines

**Fig 8:**
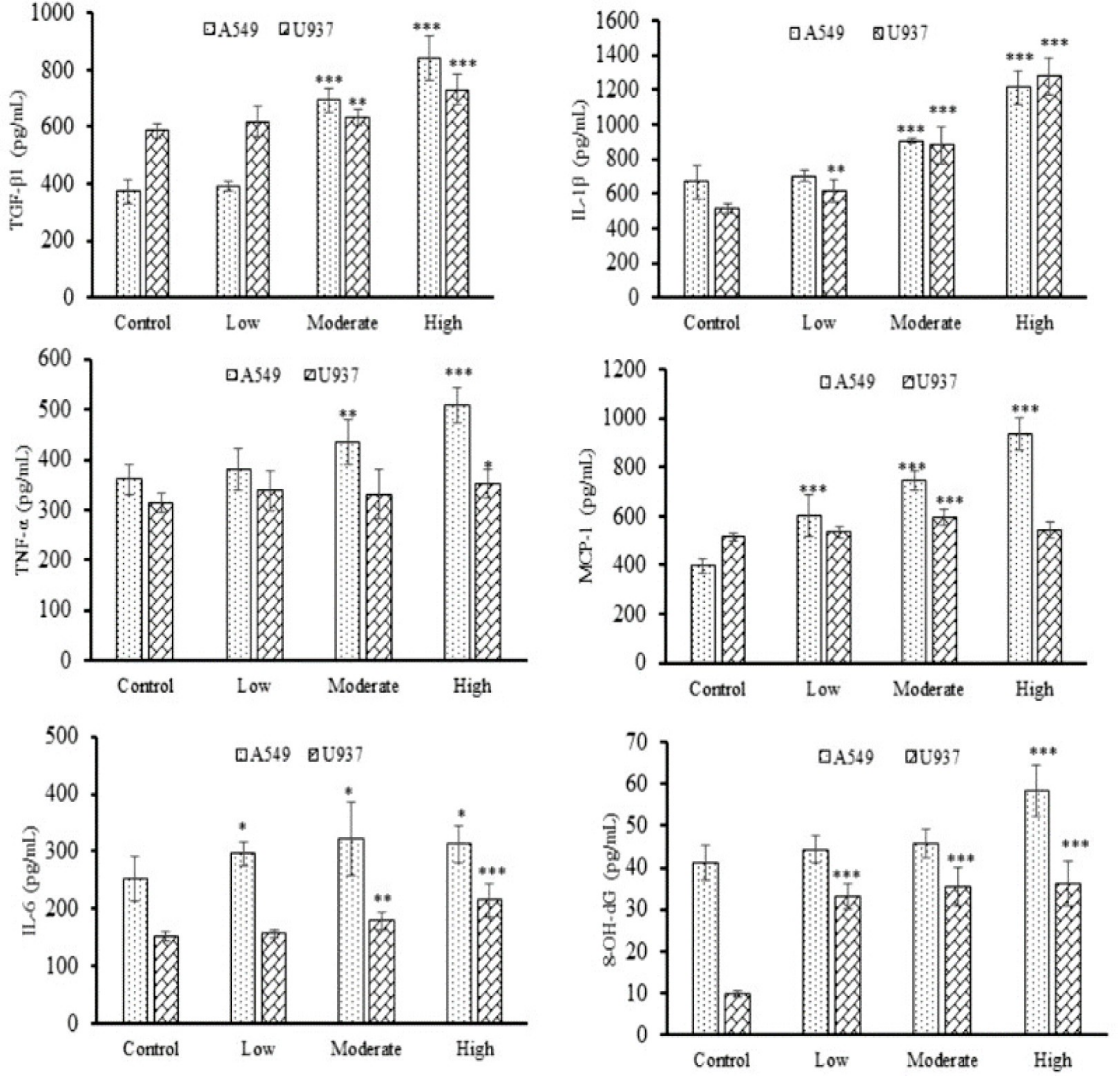
Mean data ± SD (four individual experiments in triplicate) for TGF-β1, IL-1β, TNF-α, MCP-1, IL-6, and the 8-OH-dG in A549 and U937^d^ cells lines after 48 hours of low, moderate, and high BAI-RCD samples exposure. Significantly different from the control group: *p < 0.05, **p < 0.01, ***p < 0.001; Tukey’s test.

This investigation measured representative BAI-RCD-treated A549 and U937^d^ cell lines for inflammatory cytokines to assess CWP induction. The One-way ANOVA with Tukey post hoc test was used to demonstrate statistically significant increases in TGF-β1, IL-1β, and IL-6 cytokines (p <0.001) as compared to the control (Fig 8) in both cell lines. In A549, Low BAI-RCD exposure increased MCP-1 (p < 0.001) and IL-6 (p < 0.05) against control. But other cytokines revealed a slight but insignificant increase due to low BAI-RCD besides control. In comparison, U937^d^ cells treated with high BAI-RCD demonstrated a highly significant increase (*p* < 0.001) in the concentration of TGF-β1, IL-1β, and IL-6 compared to the control. In contrast, minor but notable increased levels (*p* < 0.01 and *p* < 0.05) were encountered for TNF-α and MCP-1, respectively. Moderate BAI-RCD exposed cells also showed a significant increase (*p* < 0.01) in IL-1β and MCP-1 and (*p*< 0.05) in TNF-α and IL-6 against control. However, no cytokines were significantly increased for low BAI-RCD.

The endogenous oxidative DNA damage as a sign of genotoxicity, the 8-OH-dG, was quantified in BAI-RCD exposed A549 and U937^d^ cell lines. After 48 hours of RCD treatment, the One-way ANOVA/Tukey *post hoc* test demonstrated a significantly elevated concentration of 8-Oh-dG in A549 cells exposed to high BAI-RCD (*p* < 0.001) as compared to control (Fig 8). But low and moderate BAI-RCD did not show a significant difference in 8-OH-dG level against the control. On the other hand, a significant increase in 8-OH-dG level was reported in U937^d^ cells treated with all representative BAI-RDC doses (*p* < 0.01) against control.

## 4. Discussion

The current study aimed to identify the coal factor(s) responsible for coal miners’ lung illnesses. The impact of BAI found in respirable coal dust was the focus of the present investigation. The problem of iron-containing coal dust exposure is not confined to India. The results focused on the mean concentrations of BAI in coal samples (n = 77) differed significantly, ranging from 275 mg kg^-1^ to 9065 mg kg^-1^, and were categorized into three classes (low, moderate, and high). The variations in BAI concentration in coal revealed regional variances in coal toxicity. This can be compared with the high BAI in Pennsylvania (12149 mg kg^-1^), moderate in West Virginia (4658.8 mg kg^-1^), and low in Utah (9.6 mg kg^-1^) coal mining areas of the United States, correlated with the prevalence of the CWP. [12, 16].

The released percentage of BAI in acidic conditions against total BAI concentration after three days of incubation is 22.18%, 26.17%, and 54% of the BAI in the B3, J8, and D6 coal samples, respectively. These observations indicate that the high BAI-RCD coal sample D6 poses high geogenic exposure to the coal workers compared to low and moderate samples. The BAI stability was noted for three days for all BAI-RCD samples at pH 8.2 under condition A (incubation before heating treatment). It was found that BAI stability decreased after incubation for seven days. As the incubation period increases, the release of BAI starts declining; this signifies that BAI release capacity decreases. Furthermore, the BAI stability decreased continuously under condition B (incubation after heating), regardless of the low, moderate, or high BAI-RCD samples. Iron has to be heated to enhance its solubility since it is insoluble in water at pH 8.2 and is not bioavailable for redox reactions [12].

The comprehensive characterization of whole-coal representative samples done by the ICP-OES method indicates that the high amount of total Iron in the high BAI-RCD sample confirmed that the sample selection under the defined categories was accurate. The Ca and Si content was less in representative RCD coal samples within the usual range. Furthermore, the concentration of Si is lower, showing that the cytotoxic impact in this research is only attributable to BAI and not Si and other metals. An overall characterization of respirable mixed coal dust chemical composition can be used to identify its sources and evaluate health effects that will lead to more effective mitigation strategies [31].

Macrophages and epithelial cells are vital components of the pulmonary defence system and are involved in the pathophysiology of tissue damage caused by dust [32, 33]. We employed epithelium & macrophage cell line A549 and U937 cell lines to build a more effective in vitro model. Both cell lines work similarly after BAI-RCD exposure. D6 coal sample releases iron into the medium at a higher rate and became bioavailable for cellular uptake, exhibiting significant cytotoxicity than low and moderate BAI containing coal. A high BAI coal sample was more harmful to cause cell damage or even death and may act as a potential inducer of the events that lead to oxidative damage, inflammation, fibrosis, and CWP. Since, monocytes that are essential for the immune response and the generation of macrophages [25]. Cytotoxicity tests on monocytes along with macrophages, and epithelial cell therefore could give comprehension of the toxicological effects of coal dust respiration.

To support our investigation, previous studies [34, 35] reported the function of Iron in coal-induced cell injury by evaluating BAI and other metals from three coal mine locations in the UT, WA, and PA; and comparing the results of the findings using an in vitro A549 cell line; reported that coal from the PA had a higher BAI content and induced more toxicity.

After BAI-RCD, exposure to A549 and U937 cell lines is likely responsible for reactive oxygen species (ROS) formation and subsequent production and release of inflammatory cytokines. Iron in coal interacts with oxygen / hydrogen peroxide (O_2_/H_2_O_2_) and generates ROS when it enters cells [36]. Pulmonary macrophages can phagocytose coal dust particles, and activated macrophages can trigger a cascade of ROS generation that can cause cell injury as depicted in Fig 1 [37, 38]. Our finding revealed that BAI-RCD exposure causes stress in cells, which leads to cell damage and the production of oxidative stress indicators such as NADPH, MPO, LPO, and PC in a dose-dependent manner (low < moderate < high). Both cell lines demonstrated increased NADPH and neutrophil MPO levels in high BAI-RCD, which may be associated with pulmonary impairment. As in our study, PC level was higher in BAI-RCD samples than control, which may be more vulnerable to a lung illness. In the same context, Levine et al. [39] reported that elevated plasma protein carbonyl concentrations often indicate lung associated disease.

Oxidative stress may be mitigated by supplementing with antioxidants or activating endogenous antioxidants [40]. Antioxidant enzymes like SOD, CAT, and GSH protect cells against oxygen radicals, free radicals, and other agents that aged or kill cells [41, 42]. In the present study, a decrease (low > moderate > high) in the SOD, CAT, and GSH levels showed that these antioxidant parameters might not protect cells from the effect of BAI, which damages the cells. CAT is a powerful tool capable of degrading millions of H_2_O_2_ molecules every second. At low concentrations, H_2_O_2_ impacts cell proliferation, cell death, glucose metabolism, mitochondrial function, platelet activation, and thiol redox equilibrium [34, 43]. In this context, our study showed a low level of CAT in a high BAI-RCD sample, specifying the effect of BAI may be toxic to cells. GSH, a major cellular antioxidant known to protect the lung from oxidants [44], was significantly decreased in BAI-RCD samples compared to the control. The current study finding implies that BAI released from coal significantly affects the oxidant/antioxidant equilibrium, resulting in ROS artillery that may be linked to fundamental physiological pathways involved in the onset of inflammation and the development of CWP. However, the oxidant species formed by BAI in cells are not well understood.

The inflammatory process involves many cytokines, chemokines, and inflammatory substances. However, only a few cytokines have been extensively examined in relation to BAI, including MCP-1, TNF-, IL-1, TGF-, and IL-6, which have both pro and anti-inflammatory effects. We found elevated levels of all the cytokines in the BAI-RCD exposed A549 and U937^d^ cells, regardless of their pro or anti-inflammatory capabilities. The expression of TGF-β and IL-1 increased for both cell lines, which may trigger the inflammatory cascade. TNF-α and IL-1 are early mediators of lung inflammation [45] extensively secreted by activated macrophages and epithelial BAI-RCD exposed cells in our study. MCP-1 and IL-6 levels have been shown to rise in BAI-RCD exposed cell lines in a dose-dependent manner. In this context, High MCP-1 and IL-8 levels were shown to be substantially linked with pneumoconiosis patients and in simple and progressive massive fibrosis (PMF) [46–48]. Coal dust-exposed workers had higher levels of this cytokine than non-exposed individuals [49]. In addition, the DNA damage marker (8-OH-dG) for genotoxicity assessment was increased according to the BAI content in the coal sample. The DNA damage biomarkers showed statistically significantly higher values in the coal dust-exposed group than in the non-exposed [50]. Furthermore, our findings support previous inflammatory studies on pneumoconiosis and other lung impairments.

To summarize, BAI stimulates cytokine synthesis, irrespective of whether it is pro or anti-inflammatory. Thus, these inflammatory molecules might be surrogate indicators and legitimate targets for therapeutic intervention in controlling chronic inflammation and tissue fibrosis. However, there is little direct evidence that BAI in coal is the active compound responsible for lung injury, and the molecular mechanisms underlying coal dust pneumoconiosis and fibrosis are unclear.

The results of various experimentations from this study demonstrated that iron released from coal due to oxidations becomes bioavailable and eventually responsible for cellular toxicity. In the USA, NIOSH established a safety exposure threshold of 1 mg/m3 for coal dust for 10 hours per day. According to the findings of the current investigation, cytotoxicity can even be caused by a low BAI of 275 mg/kg. In light of these findings, we recommended that, even in cases where coal has BAI concentrations of less than ≤1000 ppm each coal mining authority adhere to the NIOSH safety standard for exposure to coal dust. However, we recognize that in order to implement safe mining practice in real-world scenario, it is crucial to define a safe level of BAI. Building on the results of our current study, we therefore suggest performing clinical trials including coal mine workers to ascertain safe concentration limits and times of exposure.

## 5. Conclusions

The current study findings indicate that BAI, the active agent, showed vast geographical differences in Indian coal and may be utilized as a predictor of coal toxicity before large-scale mining. The established in vitro model was used to assess the toxicity-causing potentials of BAI present in low, moderate and high concentrations in Indian coal as an implication for the development of CWP. As a result, mines linked with a higher risk of CWP can be categorized according to BAI releasing ability of coal. Moreover, the study findings suggest that, the dust exposure safety standards of 1mg/m^3^ should be implemented in advance. In conclusion, stakeholders can work together to create a safer and healthier environment for miners while also easing financial strains on both workers and mine owners by addressing safety issues, lowering disease burdens, and embracing innovative solutions like bioavailable iron supplementation.

## Supporting information

S1 Table Confirmation of respirable coal dust size particles (less than 10μm) by Particle Size Analyzer (PSA). D—Value (D_**10**_, D_**50**_ & D_**90**_) are the interceptsfor 10%, 50%, and 90% of the cumulative mass.
(DOCX)

S2 Table Estimation of BAI in all the coal samples (n = 77) of different mines region of India. (DOCX)

S1 Fig Graphical representation of Particle Size Analysis (PSA) of High BAI containing respirable coal dust (less than 10μm). (DOCX)

S2 Fig Graphical representation of Particle Size Analysis (PSA) of Moderate BAI containing respirable coal dust (less than 10μm). (DOCX)

S3 Fig Graphical representation of Particle Size Analysis (PSA) of Low BAI containing respirable coal dust (less than 10μm). (DOCX)

S4 Fig Standard graph obtained from different iron standards for measurement of BAI in coal samples by quantichrom kit method. Slope y = 4379x, R^2^ = 0.982 indicating fitted regression line.
(DOCX)

S5 Fig Demonstrates standardization graph of oxidative stress parameters (NADPH, MPO, MDA, PC, SOD, CAT, and GSH) in A549 & U937^d^ cell lysate using the kit method. Standardized regression line equations of Oxidative stress parameters will be used to calculate their level in A549 & U937^d^ cell lysate after exposure to low, moderate, and high BAI-containing coal dust samples.
(DOCX)

S6 Fig Demonstrates standardization graphs of human cytokine a) TNF—alpha b) IL– 6 c) MCP-1 d) TGF - 1B e) IL—1 B using the kit method. Standardized regression line equations were used to calculate the respective cytokines in the BAI-RCD exposed cell culture supernatant.
(DOCX)

S7 Fig Demonstrates standardization graph of DNA damage marker 8-hydroxideoxyguanosine (8-OHdG) using the kit method. A standardized regression line equation was used to calculate the 8-OHdG in the cell culture supernatant after exposure with low, moderate, and high BAI-containing coal dust samples.
(DOCX)
