# Supplementary material for: In vitro toxicity assessment of bioavailable iron in coal varieties of Central India
Source: PLoS One. 2024 Sep 19;19(9):e0309237. doi: 10.1371/journal.pone.0309237 (PMC11412545; doi:10.1371/journal.pone.0309237)
Supplement: S1 Table — D—Value (D10, D50 & D90) are the interceptsfor 10%, 50%, and 90% of the cumulative mass. (DOCX) [file pone.0309237.s001.docx]

S1 Table. Confirmation of respirable coal dust size particles (less than 10μm) by Particle Size Analyzer (PSA)

| Sr. No. | Coal type | Description | Report |
| --- | --- | --- | --- |
| 1 | High BAI coal | 212μm Sample +5 min. cup milled +sieved through 25μm sieve | Highest:205 μm  Less than 10μm: 75%  Median: 4.204 μm  Mean: 9.573 μm  D_10_:1.006 μm  D_50_: 4.204 μm  D_90_: 18.828 μm |
| 2 | Moderate BAI coal | 212μm Sample +5 min. cup milled +sieved through 25μm sieve | Highest:129.746 μm  Less than 10μm: 75%  Median: 4.275μm  Mean: 8.066μm  D_10_:1.043μm  D_50_: 4.275μm  D_90_: 17.765μm |
| 3 | Low BAI coal | 212μm Sample +5 min. cup milled +sieved through 25μm sieve | Highest:41.029μm  Less than 10μm:75%  Median: 5.056μm  Mean: 7.323μm  D_10_: 1.174μm  D_50_:5.056 μm  D_90_: 15.561μm |

D-Values (D_10_, D_50_ & D_90_) are the intercepts for 10%, 50%, and 90% of the cumulative mass.

The above table demonstrated the confirmation of respirable coal dust preparation (less than 10µm) from the original 212 µm coal dust sample by Laser particle size analyzer. The particle size was confirmed in the selected categorized BAI content three coal samples indicating that all the samples had achieved 75% of particles were less than 10μm. High BAI coal showed the mean = 9.573μm of all the coal particles size, the intercept (D_50_) of cumulative mass of coal sample = 4.204sμm indicating maximum coal particles had attained the respirable coal dust size (i.e., less than 10μm) likewise in the moderate and Low BAI coal samples. Confirmation of respirable coal dust size particles in all the selected three samples with high, moderate, and low BAI content was necessary because these samples were used for further experiments.
