# Supplementary material for: In vitro toxicity assessment of bioavailable iron in coal varieties of Central India
Source: PLoS One. 2024 Sep 19;19(9):e0309237. doi: 10.1371/journal.pone.0309237 (PMC11412545; doi:10.1371/journal.pone.0309237)
Supplement: S2 Table — (DOCX) [file pone.0309237.s002.docx]

**S2 Table. Estimation of BAI in all the coal samples (n=77) of different mines regions of India.**

| **MINES REGION** | **SAMPLE CODE** | **BAI (ppm)** |
| --- | --- | --- |
| **Mine Region 1** | M 1 | 2825 |
|  | A 2 | 3625 |
|  | A 3 | 3475 |
|  | A 4 | 4125 |
|  | A 5 | 3550 |
|  | A 6 | 3025 |
|  | A 7 | 3375 |
|  | A 8 | 4250 |
|  | A 9 | 3575 |
|  | A 10 | 2750 |
|  | A 11 | 3575 |
|  | A 12 | 3722 |
|  | A 13 | 3125 |
|  | A 14 | 2277 |
|  | A 15 | 3675 |
|  | A 16 | 4291 |
| **Mine Region 2** | B 1 | 1000 |
|  | B 2 | 600 |
|  | B 3 | 275 |
|  | B 4 | 3800 |
|  | B 5 | 1525 |
|  | B 6 | 1100 |
| **Mine Region 3** | C 1 | 2675 |
|  | C 2 | 2671 |
|  | C 3 | 1525 |
|  | C 4 | 875 |
|  | C 5 | 1375 |
|  | C 6 | 375 |
| **Mine Region 4** | D 1 | 3925 |
|  | D 2 | 2719.8 |
|  | D 3 | 3725 |
|  | D 4 | 2190 |
|  | D 5 | 7180 |
|  | D 6 | 9065 |
|  | D 7 | 1270 |
|  | D 8 | 2540 |
| **Mine Region 5** | E1 | 1625 |
|  | E2 | 775 |
|  | E3 | 475 |
|  | E4 | 345 |
|  | E5 | 875 |
|  | E6 | 500 |
|  | E7 | 575 |
|  | E8 | 625 |
|  | E9 | 3025 |
|  | E10 | 4875 |
| **Mine Region 6** | F1 | 3850 |
|  | F2 | 3200 |
|  | F3 | 1800 |
|  | F4 | 3125 |
|  | F5 | 1800 |
|  | F6 | 3460 |
|  | F7 | 2802 |
|  | F8 | 3325 |
| **Mine Region 7** | G1 | 3225 |
|  | G2 | 3330 |
|  | G3 | 4200 |
|  | G4 | 4466 |
| **Mine Region 8** | H1 | 1700 |
|  | H 2 | 2275 |
|  | H 3 | 2585 |
|  | H4 | 1525 |
| **Mine Region 9** | I1 | 2375 |
|  | I2 | 2950 |
|  | I3 | 4905 |
|  | I4 | 2450 |
|  | I5 | 2575 |
| **Mine Region 10** | J 1 | 1850 |
|  | J 2 | 800 |
|  | J 3 | 2825 |
|  | J 4 | 2125 |
|  | J 5 | 2625 |
|  | J 6 | 1975 |
|  | J 7 | 3125 |
|  | J 8 | 4650 |
|  | J 9 | 2175 |
|  | J 10 | 2900 |
