# Supplementary material for: In vitro toxicity assessment of bioavailable iron in coal varieties of Central India
Source: PLoS One. 2024 Sep 19;19(9):e0309237. doi: 10.1371/journal.pone.0309237 (PMC11412545; doi:10.1371/journal.pone.0309237)
Supplement: S2 Fig — (DOCX) [file pone.0309237.s004.docx]

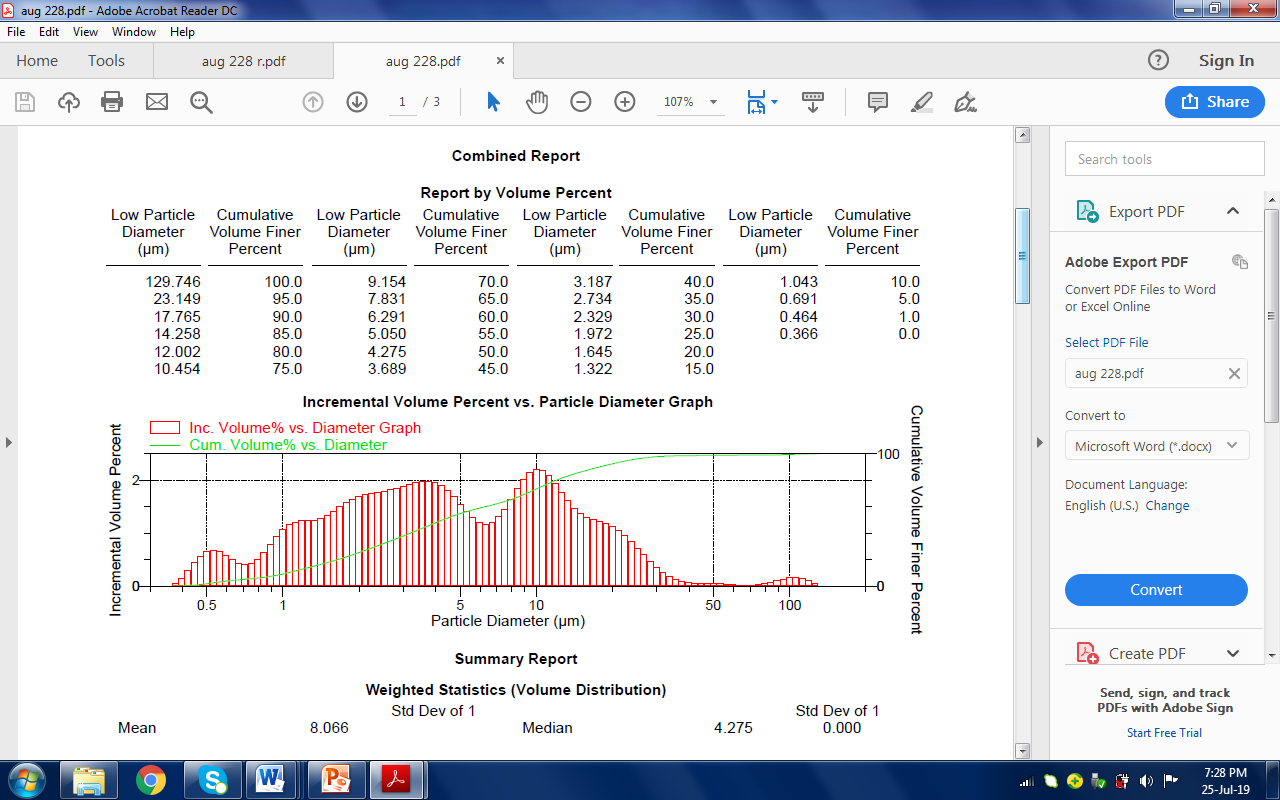


**S2 Fig.** **Graphical representation of Particle Size Analysis (PSA) of Moderate BAI containing respirable coal dust (less than 10μm).**

Moderate BAI containing coal showed the mean = 8.066μm of all the coal particles size, the intercept (D_50_) of cumulative mass of coal sample = 4.275μm indicating maximum coal particles (75%) had attained the respirable coal dust size (i.e., less than 10μm).
