# Supplementary material for: In vitro toxicity assessment of bioavailable iron in coal varieties of Central India
Source: PLoS One. 2024 Sep 19;19(9):e0309237. doi: 10.1371/journal.pone.0309237 (PMC11412545; doi:10.1371/journal.pone.0309237)
Supplement: S3 Fig — (DOCX) [file pone.0309237.s005.docx]

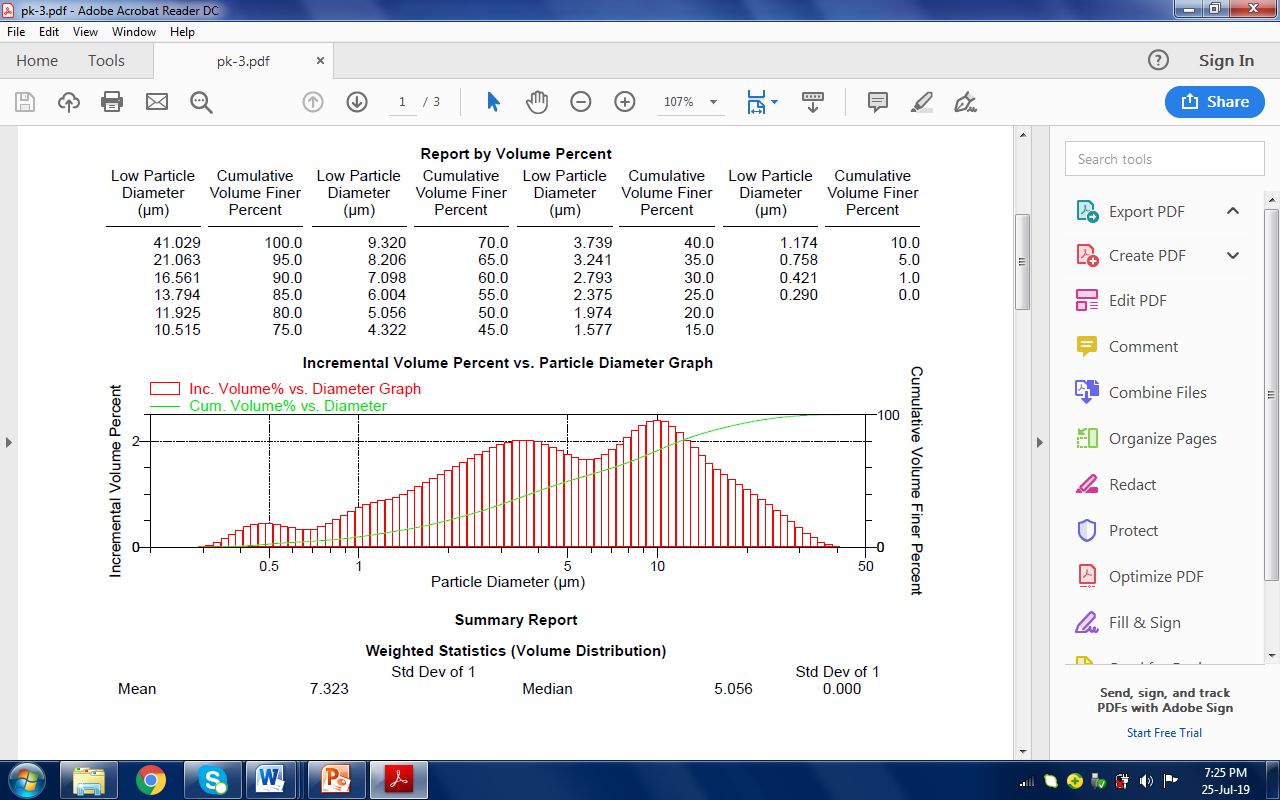


**S3 Fig.** **Graphical representation of Particle Size Analysis (PSA) of Low BAI containing respirable coal dust (less than 10μm).**

Low BAI containing coal showed the mean = 7.323μm of all the coal particles size, the intercept (D_50_) of cumulative mass of coal sample = 5.056μm indicating maximum coal particles (75%) had attained the respirable coal dust size (i.e., less than 10μm).
