# Supplementary material for: In vitro toxicity assessment of bioavailable iron in coal varieties of Central India
Source: PLoS One. 2024 Sep 19;19(9):e0309237. doi: 10.1371/journal.pone.0309237 (PMC11412545; doi:10.1371/journal.pone.0309237)
Supplement: S4 Fig — Slope y = 4379x, R2 = 0.982 indicating fitted regression line. (DOCX) [file pone.0309237.s006.docx]

**
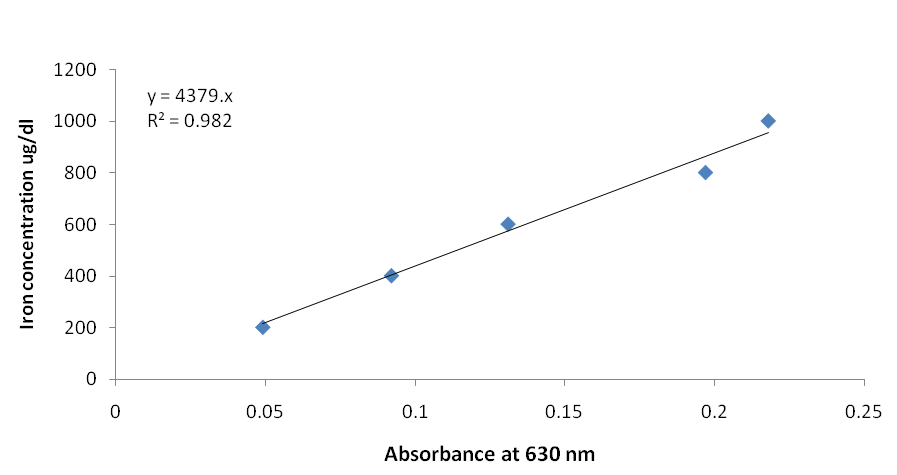
**

**S4 Fig. Standard graph obtained from different iron standards for measurement of BAI in coal samples by quantichrom kit method.** Slope y = 4379x, R^2^ = 0.982 indicating fitted regression line
