# Supplementary material for: In vitro toxicity assessment of bioavailable iron in coal varieties of Central India
Source: PLoS One. 2024 Sep 19;19(9):e0309237. doi: 10.1371/journal.pone.0309237 (PMC11412545; doi:10.1371/journal.pone.0309237)
Supplement: S5 Fig — Standardized regression line equations of Oxidative stress parameters will be used to calculate their level in A549 & U937d cell lysate after exposure to low, moderate, and high BAI-containing coal dust samples. (DOCX) [file pone.0309237.s007.docx]

**
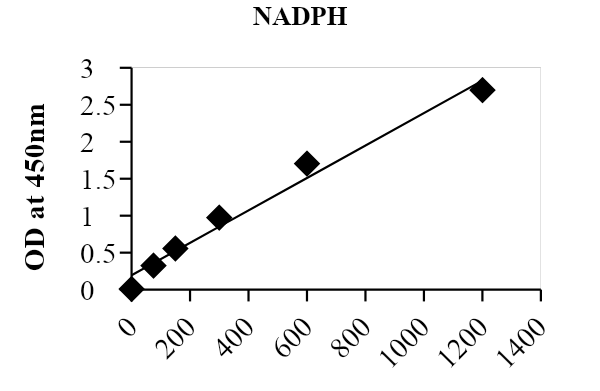
**

**
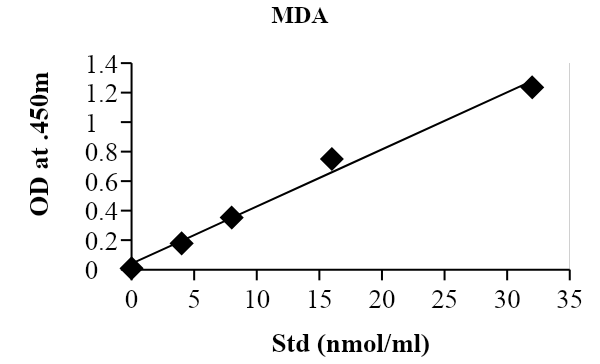
**

**
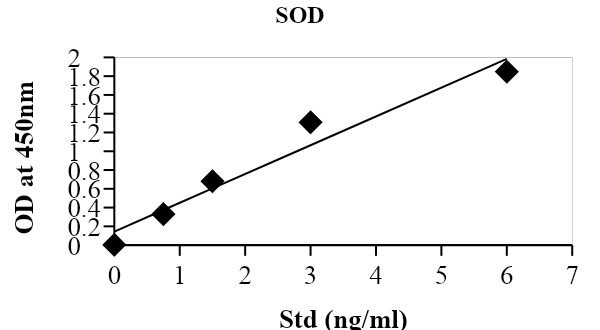
**

**S5 Fig. Demonstrates standardization graph of oxidative stress parameters (NADPH, MPO, MDA, PC, SOD, CAT, and GSH) in A549 & U937^d^ cell lysate using the kit method.**  Standardized regression line equations of oxidative stress parameters will be used to calculate their level in A549 & U937d cell lysate after exposure to low, moderate, and high BAI-containing coal dust samples.

**
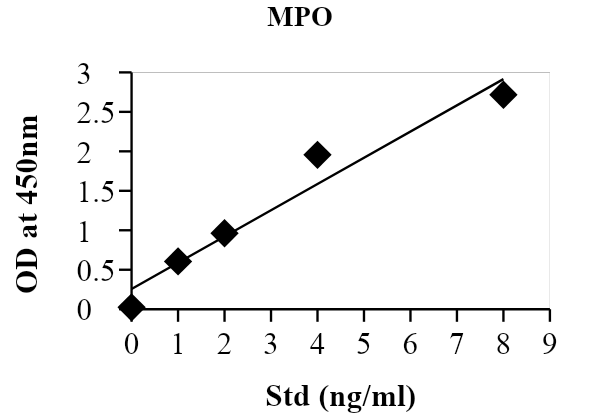
**

**
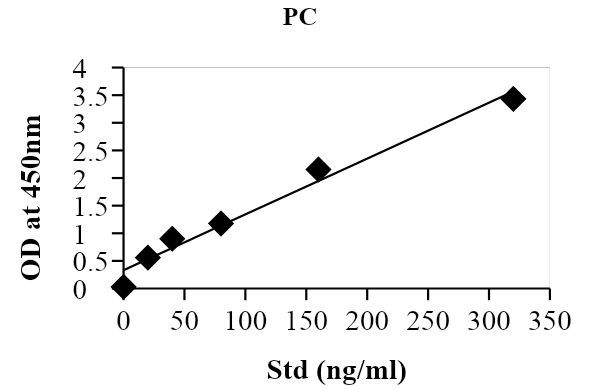
**

**
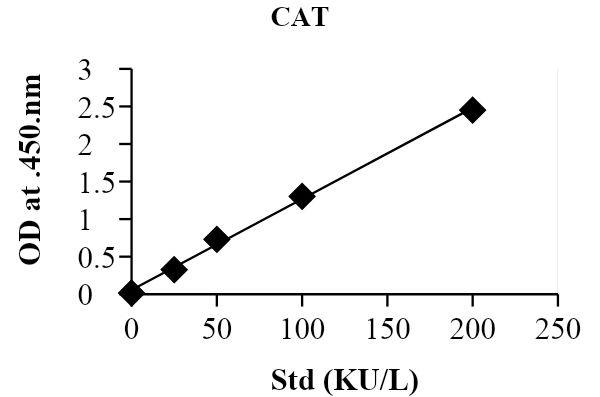
**
