# Supplementary material for: In vitro toxicity assessment of bioavailable iron in coal varieties of Central India
Source: PLoS One. 2024 Sep 19;19(9):e0309237. doi: 10.1371/journal.pone.0309237 (PMC11412545; doi:10.1371/journal.pone.0309237)
Supplement: S6 Fig — Demonstrates standardization graphs of human cytokine a) TNF—alpha b) IL– 6 c) MCP-1 d) TGF - 1B e) IL—1 B using the kit method. Standardized regression line equations were used to calculate the respective cytokines in the BAI-RCD exposed cell culture supernatant. (DOCX) [file pone.0309237.s008.docx]

| 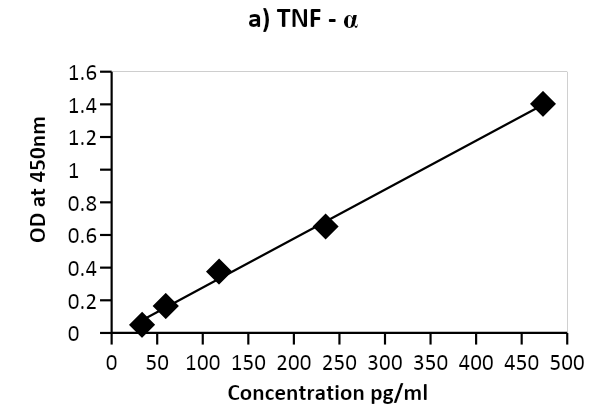 | 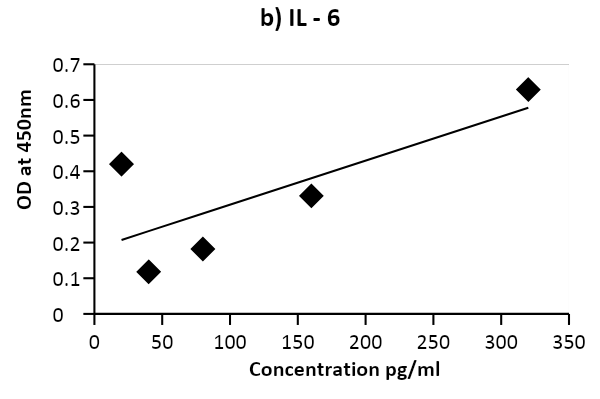 |
| --- | --- |
| 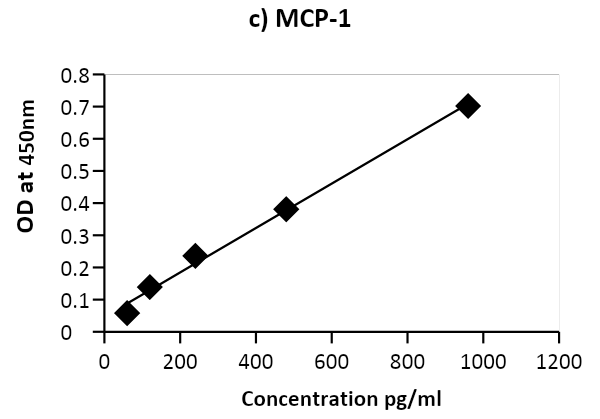 | 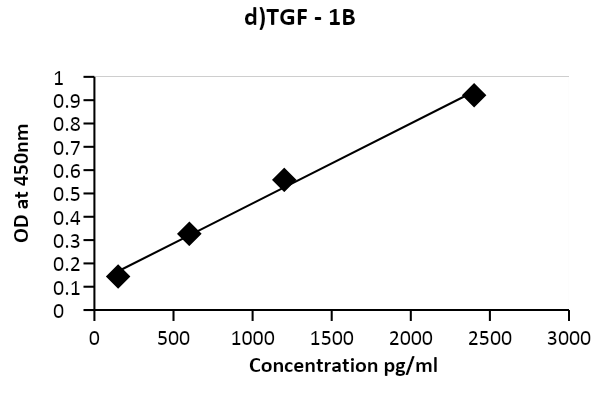 |
| 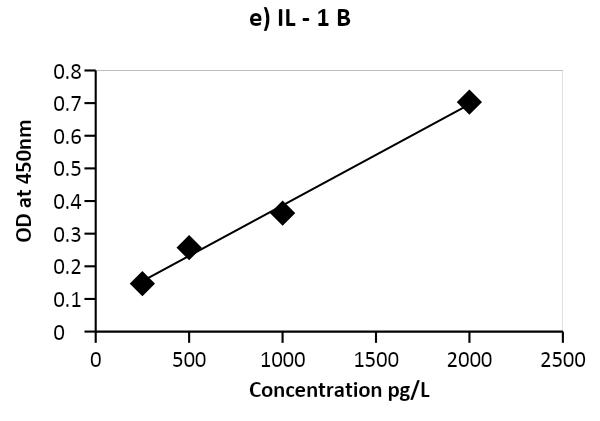 |  |

**S6 Fig. Demonstrates standardization graphs of human cytokine a) TNF - alpha b) IL - 6 c) MCP-1 d) TGF - 1B e) IL - 1 B using the kit method.** Standardized regression line equations were used to calculate the respective cytokines in the BAI-RCD exposed cell culture supernatant.
