# Supplementary material for: In vitro toxicity assessment of bioavailable iron in coal varieties of Central India
Source: PLoS One. 2024 Sep 19;19(9):e0309237. doi: 10.1371/journal.pone.0309237 (PMC11412545; doi:10.1371/journal.pone.0309237)
Supplement: S7 Fig — A standardized regression line equation was used to calculate the 8-OHdG in the cell culture supernatant after exposure with low, moderate, and high BAI-containing coal dust samples. (DOCX) [file pone.0309237.s009.docx]

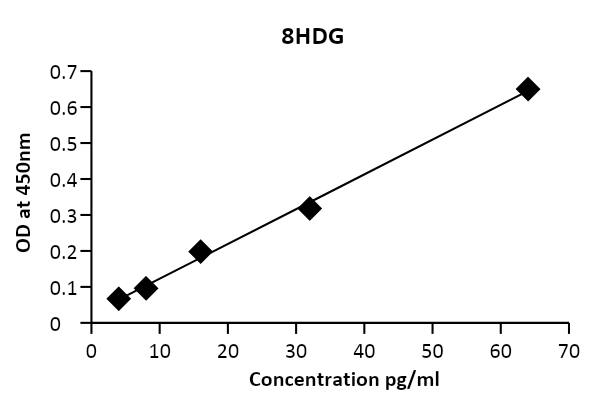


**S7 Fig**. **Demonstrates standardization graph of DNA damage marker 8-hydroxideoxyguanosine (8-OHdG ) using the kit method.** A standardized regression line equation was used to calculate the 8-OHdG in the cell culture supernatant.
